# Lengths of Stay and Stopover Strategies of Western Sandpipers During Migration at Two Sites in British Columbia, Canada

**DOI:** 10.1002/ece3.70739

**Published:** 2025-01-11

**Authors:** Anne L. Blondin, Mark C. Drever, Scott A. Flemming, Wendy E. Easton, Mark Maftei, Yuri Zharikov, Nils Warnock, Erica Nol

**Affiliations:** ^1^ Trent University Peterborough Ontario Canada; ^2^ Environment and Climate Change Canada Delta British Columbia Canada; ^3^ Raincoast Education Society Ucluelet British Columbia Canada; ^4^ Pacific Rim National Park Reserve Parks Canada Ucluelet British Columbia Canada; ^5^ Audubon Canyon Ranch Stinson Beach California USA

## Abstract

An accurate estimate of length of stay is necessary to derive passage population size for birds using a migration stopover site. In this study, we used VHF tags and a Motus automated telemetry array to estimate the length of stay of 385 Western Sandpipers ( *Calidris mauri* ) migrating through two stopover sites in British Columbia, Canada (Tofino and Fraser River Estuary) over the course of seven migration periods (three northward and four southward) from 2018 to 2021. The average length of stay of Western Sandpipers at the Tofino site on the west coast of Vancouver Island varied from 2 to 6 days and was shorter than the length of stay at the Fraser River Estuary, where the average length of stay varied from 4 to 8 days. At both sites, adult birds had shorter stopovers during southward migration, juvenile birds had longer stopovers compared to adults, and birds with lower mass at capture had longer stopovers. Morphology of adults and juveniles varied between the two sites. Birds captured at Tofino had shorter tarsi, as well as higher mass during southward migration compared to Western Sandpipers captured at the Fraser River Estuary. We also assessed prey availability at the two stopover sites, and we found that invertebrate density was greater in Tofino compared to the Fraser River Estuary during northward migration. Variation in minimum stopover length and morphology between sites suggests that individuals from different overwintering populations may use different routes along the west coast of North America. Western Sandpipers stopping at Tofino have a shorter length of stay during both migration periods and arrive heavier during southward migration, characteristics typical of “hop” migrants who travel shorter distances between stopover sites. Different stopover sites offer a unique set of site characteristics used by birds exhibiting varying migration strategies, highlighting the importance of conserving a diversity of migration stopover locations.

## Graphical abstract

Stopover duration (length of stay, LOS) is a key variable in avian migration ecology that reflects behavioral decisions to obtain fuel or continue travel between breeding and non‐breeding areas. We find the LOS of Western Sandpipers differs between two important stopover sites on the Pacific Coast of Canada, reflecting different migration strategies that depend on location along the flyway.

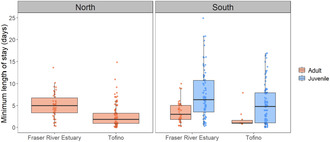

## 1 Introduction

Most species of North American shorebirds depend on stopover sites to rest and refuel during long migratory journeys between breeding grounds and non‐breeding grounds (Warnock and Bishop 1998; Warnock 2010). A single stopover site can be used by millions of shorebirds during migratory periods, and these congregations can represent large proportions of global populations (Drever et al. 2014). Habitat loss or degradation at these key stopover sites can have detrimental effects on a species population (Studds et al. 2017). Shorebird populations in North America have experienced a net decline of 37% since 1970, and these losses are accelerating (Rosenberg et al. 2019; Smith et al. 2023). Thus, understanding how shorebirds use stopover sites and maintaining a network of these critical areas is imperative for shorebird conservation efforts (Studds et al. 2017; Duan et al. 2022; Wang et al. 2022).

Length of stay is a key metric required to accurately estimate the total passage population of birds using a migration stopover site (Bishop, Meyers, and Furtsch McNeley 2000; Farmer and Durbian 2006; Macdonald et al. 2021; Neima et al. 2022). The total number of birds using such sites is difficult to assess due to the continuous flow of birds throughout the migration period (Bishop, Meyers, and Furtsch McNeley 2000). Understanding length of stay and its seasonal, annual, or interspecific variations is further complicated by a variety of factors. Habitat characteristics (e.g., prey availability or predation danger; Ydenberg et al. 2002; Lank et al. 2003; Taylor et al. 2007), individual physiology (e.g., age, body condition, sex; Anderson et al. 2019; Herbert, Mizrahi, and Taylor 2022), environmental conditions (e.g., wind, drought, human disturbance; Canham et al. 2021; Anderson et al. 2021; Herbert, Mizrahi, and Taylor 2022), and temporal variables (e.g., migration season, stopover arrival time) can all influence length of stay (Butler, Shepherd, and Lemon 2002; Lank et al. 2003; Taylor et al. 2007). Therefore, understanding how length of stay can vary in space and time is important in determining the ecological processes that underlie changes in shorebird abundance at stopover sites.

Shorebirds generally exhibit three patterns of migration depending on the distance traveled between stopover sites and energetic requirements to make the flights (Piersma 1987; Warnock 2010). “Hop” migrants travel short to intermediate distances between stopover sites, have shorter lengths of stay, lower fueling rates, and depart to subsequent locations on their migratory route with smaller fuel stores; characteristics that are also associated with an energy‐conserving strategy. Conversely, “jump” migrants travel long distances between stopover sites, have longer lengths of stay, higher fueling rates, and depart to subsequent locations with larger fuel stores; behaviors associated with a time‐conserving migration strategy. “Skip” migrants travel intermediate distances between stopovers and exhibit migration behaviors somewhere between “hop” and “jump” migrants (Piersma 1987; Alerstam and Lindström 1990; Warnock 2010; Henkel and Taylor 2015; Anderson et al. 2019).

Western Sandpipers ( *Calidris mauri* ) are an abundant shorebird species along the Pacific Americas Flyway (Page, Stenzel, and Kjelmyr 1999), numbering 3.0–4.5 million birds based on surveys conducted in the 1990s (Bishop, Meyers, and Furtsch McNeley 2000), and their populations are decreasing on the Pacific coast of North America (Hope et al. 2019; Canham et al. 2021; Warnock et al. 2021). The length of stay of Western Sandpipers during pre‐breeding migration (hereafter: northward) has been calculated at numerous sites along the Pacific Americas Flyway, such as the Fraser River Estuary, using mark‐recapture (re‐sighting), ground and aerial telemetry, and hydrological modeling methods (Butler, Kaiser, and Smith 1987; Iverson et al. 1996; Warnock and Bishop 1998; Warnock, Takekawa, and Bishop 2004; Drever and Hrachowitz 2017). Fewer studies have assessed Western Sandpipers' length of stay and factors influencing stopover duration during post‐breeding migration (hereafter: southward), including differences between adults and juveniles (Ydenberg et al. 2004; Hope et al. 2021). In addition to large estuaries in British Columbia, Western Sandpipers also stop at smaller sites on the west coast of Vancouver Island during northward and southward migration (Drever et al. 2016), where relatively little information is known about their length of stay.

**FIGURE 1:**
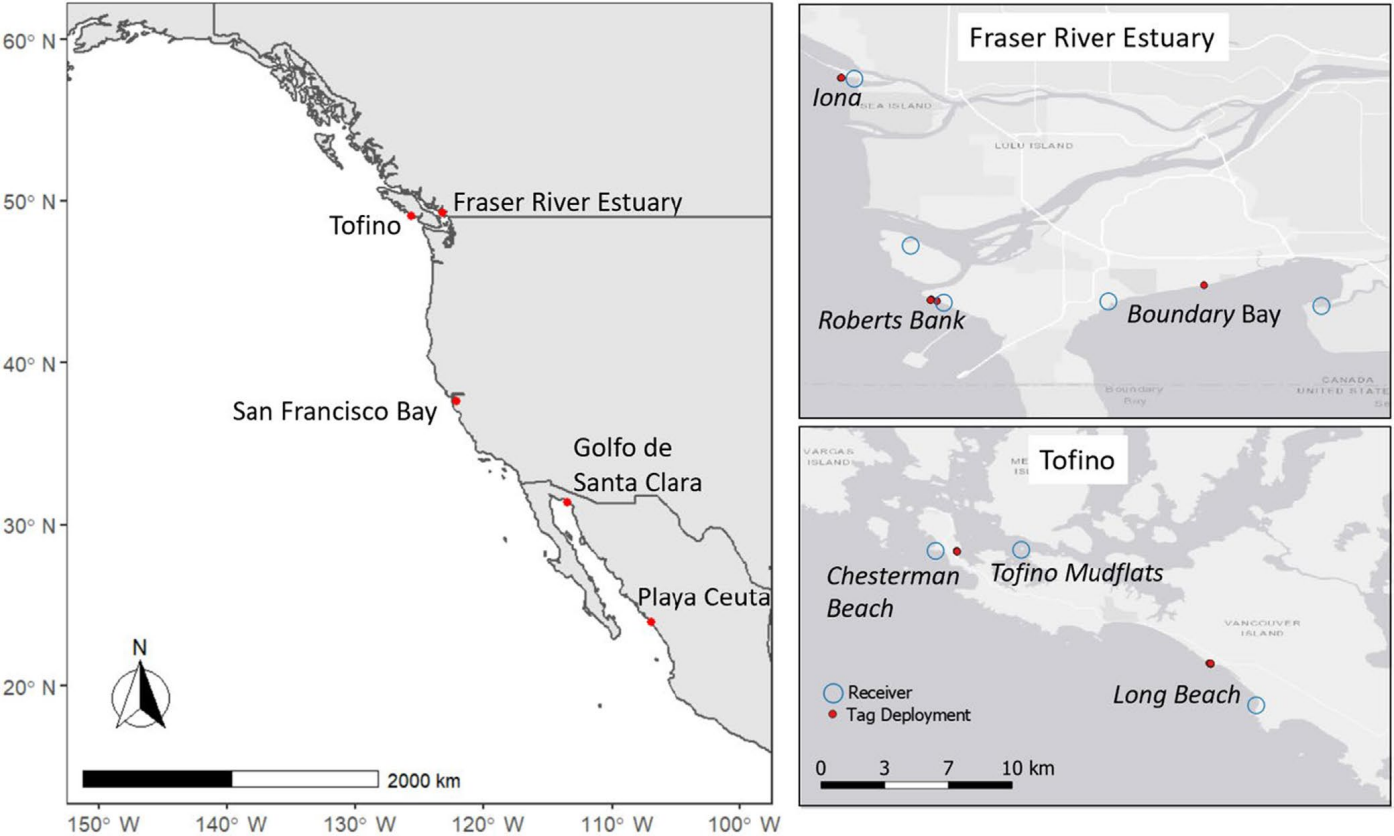
Capture sites (red points) for Western Sandpipers along the Pacific Americas Flyway (left map). Motus receiving stations (blue circles) and capture sites (red points) are located within the Fraser River Estuary (top right map) and Tofino (bottom right map) stopover study areas.

We used an automated radio‐telemetry network, the Motus Wildlife Tracking System (Taylor et al. 2017), to assess the length of stay of Western Sandpipers at two important migratory stopover sites in British Columbia, Canada: the Fraser River Estuary situated near Vancouver, British Columbia, and the Tofino Mudflats on the west coast of Vancouver Island, British Columbia (Figure 1). The purpose of this study was to answer two main questions: (1) What is the length of stay of Western Sandpipers at the Fraser River Estuary and Tofino stopover sites, and (2) What variables affect their length of stay and migration strategies? We examined the relationship between length of stay and stopover site location, initial capture site location, migration season, age, sex, and body mass. We compared the length of stay during the same migration periods at two sites that differed in habitat composition to examine how site attributes may affect the stopover behavior of migrating sandpipers. We also assessed the abundance of prey (benthic invertebrates) at each site as an indicator of site quality, which might help to explain differences in stopover behavior at our study sites. Sandpipers appear to have longer stopovers at higher quality sites (Herbert, Mizrahi, and Taylor 2022), i.e., “jump” behavior, and juvenile sandpipers may shorten their length of stay during years with low prey density (Anderson et al. 2021), i.e., “hop” behavior. To explore migration strategies during different migrations, we compared the length of stay of adult Western Sandpipers between northward and southward migration periods. Since time‐conserving strategies are often associated with longer lengths of stay, such that birds can acquire large fuel loads to increase flight distance between stops and shorten the overall migration time (Piersma 1987; Warnock 2010), we predicted Western Sandpipers would have longer stopovers during northward migration, as they would be under greater time constraints to reach their Arctic breeding grounds. We also examined relationships between length of stay and age class during southward migration. Juvenile sandpipers have no prior migration experience, and they may use stopover habitats differently and are potentially exposed to different environmental conditions during migration than adult birds (Hope et al. 2011; Linhart et al. 2023). Juvenile Western Sandpipers migrate approximately 1 month later than adults, concurrently with Peregrine Falcons ( *Falco peregrinus* ), and they may adopt different migration strategies in response to this heightened predation risk (Hope et al. 2011). Since juvenile sandpipers can have both longer (Mann et al. 2017; Linhart et al. 2023) and shorter (Hope et al. 2011) stopovers than adult birds depending on context, we therefore did not predict a direction of effect for the two age classes. Understanding how the length of stay of sandpipers can vary among sites and seasons will inform the estimation of the total passage population of shorebirds on the Pacific Flyway and guide conservation decisions related to the importance of primary versus smaller satellite sites for migration (Linhart et al. 2023).

## 2 Methods

### 2.1 Study Areas

The Fraser River Estuary (49.095, −123.125) (Figure 1) is situated approximately 15 km south of the City of Vancouver in the Lower Mainland region of British Columbia, Canada. This site extends over approximately 22,000 ha at low tide and comprises three large intertidal mudflat habitats (Sturgeon Bank, Roberts Bank, Boundary Bay) and is mostly made up of sand, silt, and clay (Drever et al. 2014). This area receives significant freshwater input from the Fraser River, creating ideal conditions for intertidal biofilm, an important food source for migrating Western Sandpipers (Kuwae et al. 2008; Schnurr et al. 2020). Industrial activity on and around the tidal mudflats includes a major shipping port, a ferry terminal, a sewage treatment plant, and river training dikes that stretch perpendicular from the shoreline into the Strait of Georgia (Atkins, Tidd, and Ruffo 2016; Drever et al. 2023). The Fraser River Estuary is a stopover site of hemispheric importance (WHSRN 2019a, 2019b), and it is estimated that up to ~50% of Western Sandpiper populations and 50% of Pacific Dunlin ( *Calidris alpina pacifica* ) populations in the Pacific Americas Flyway use the Roberts Bank mudflat, where they exploit an abundance of invertebrates and intertidal biofilm (Kuwae et al. 2008; Drever et al. 2014; Hobson et al. 2022).

The Tofino stopover site is situated on the west coast of Vancouver Island within the District of Tofino and Pacific Rim National Park Reserve (49.100, −125.852) (Figure 1) and contains approximately 2630 ha of exposed mudflats and sandflats (Government of British Columbia n.d.). Collectively, these habitats are recognized as the Tofino Wah‐nah‐jus Hilth‐hoo‐is Mudflats and include Chesterman Beach, Long Beach, and the mudflats along Browning Passage on the north side of the Esowista Peninsula. Chesterman Beach and Long Beach are beach habitats that are exposed to the open ocean, and Long Beach is located within the boundaries of the Pacific Rim National Park Reserve. The Tofino mudflats are a biologically diverse tidal mudflat complex of sheltered tidal channels, streams, marshes, and eelgrass beds, and peak counts during northward migration can see over 40,000 Western Sandpipers (Drever et al. 2016). The Tofino Mudflats have been designated as a locally important site for migrating shorebirds (WHSRN 2019a, 2019b) and is being considered for an elevated conservation status by WHSRN given very high peak counts in 2019 to 2021 (Maftei, unpublished data).

### 2.2 Shorebird Captures

We captured Western Sandpipers using mist‐nets during northward and southward migration seasons from 2018 to 2021. Capture efforts occurred during periods of peak shorebird migration, and the timing of mist‐netting sessions varied across the two study sites, seasons, and ages (adults and juveniles; Table 1). For northward migration of adult birds, we conducted captures during late April in the Fraser River Estuary and early May in Tofino. For southward migration, captures in the Fraser River Estuary were done in mid‐July for adults and mid‐August for juveniles. Southward captures in Tofino for both ages continued intermittently from late July through September. Northward shorebird captures did not occur in the Fraser River Estuary in spring 2020 due to COVID‐19 restrictions.

**TABLE 1:** Capture dates and the number of Western Sandpipers tagged with VHF radio transmitters in the Fraser River Estuary and Tofino stopover sites from 2018 to 2021.

Year	Northward				Southward			
	Fraser River Estuary		Tofino		Fraser River Estuary		Tofino	
	Tags deployed	Capture dates	Tags deployed	Capture dates	Tags deployed	Capture dates	Tags deployed	Capture dates
2018	0	—	0	—	0	—	23	Aug 08 to Sep 06
2019	15	Apr 25	34	May 01 to 07	0	—	36	Aug 01 to Sep 10
2020	0	—	37	May 01 to 08	78	Jul 11 to Aug 27	43	Jul 27 to Aug 22
2021	30	Apr 23 to 29	32	May 03 to 16	56	Jul 13 to Aug 24	11	Jul 20 to Sep 16

Upon capture, we banded each bird with a uniquely coded government‐issued metal band, and we recorded mass (g) and culmen (mm). Tarsus (mm) was not always recorded at capture: tarsus was not recorded at the Fraser River Estuary in 2021 during northward migration and was not recorded in later years at Tofino, resulting in varying sample sizes for this measurement. Wing chord (mm) was recorded at the Tofino capture sites, whereas wing chord (mm) or flattened wing (mm) were recorded in the Fraser River Estuary following methods in Prater et al. (1977). When possible, we assigned sex based on bill length (Sandercock 1998; Stein et al. 2008). We aged birds (juvenile vs. adult) based on the color of the inner medial covert feathers (O'Hara et al. 2006).

We attached small, digitally encoded Very High Frequency (VHF) nanotags to the skin on the backs of the shorebirds using cyanoacrylate adhesive. Feathers were trimmed to expose the skin just above the uropygial gland where the nanotags were attached. We used Lotek nanotag models NTQB2‐3‐2 (Lotek, Newmarket, Ontario, Canada), with a burst rate of 13.7 s and weighing 0.66 g (ranging 2.2% to 3.7% of body mass of Western Sandpipers [18 to 30 g]). We deployed a total of 395 tags from 2018 to 2021 in Tofino and Fraser River Estuary (Table 1). Additionally, collaborators deployed tags on birds ~1300 and ~3200 km south of our main study sites in California and Mexico (Figure 1) to obtain length of stay estimates unbiased by handling time at capture sites (Warnock and Bishop 1998).

### 2.3 Automated Radiotelemetry

We used the Motus Wildlife Tracking System (“Motus”; Taylor et al. 2017), a collaborative research network that uses automated telemetry, to determine the length of stay of shorebirds during migration stopover. The Motus system tracks wildlife using digitally encoded nanotags that are detected by stationary automated receivers that continuously scan for signals within a detection range of ~500 m to 15 km, depending on topography and antennae type (Taylor et al. 2017). The Fraser River Estuary and Tofino stopovers are approximately 200 km apart, and therefore the Motus detection ranges do not overlap between the two stopover sites in this study. Nanotags operated on a single frequency (166.380 mHZ), and individual tags were identified by a unique combination of sequence code and burst rates (Taylor et al. 2017). Motus receivers were strategically placed at both stopover sites in areas where shorebirds are known to congregate to feed and roost, and receiver arrays provided good spatial coverage at both sites (Figure 1). The Fraser River Estuary study began with one receiving station in 2018, and the area had seven stations by 2021. The Tofino stopover site consistently maintained three receiving stations from 2018 to 2021. The difference in the final number of towers at each stopover site was due to the overall size (Tofino being smaller) and characteristics of the two sites. Here, we refer to the receiving stations located within the two study stopover sites as the “project array.”

### 2.4 Data Cleaning

Telemetry detection data were downloaded from the Motus database using R software (v 4.3.1; R Core Team 2023) following guidelines from the R Motus Book (Crewe, Chrysler, and Taylor 2018). The data were cleaned using Motus' *filterByActivity* function (Crewe, Chrysler, and Taylor 2018). Cleaned data were further filtered to include detections by receiving stations located in the study areas. Filtered data were cleaned a second time by filtering detections with a run length (number of continuous detections) < 4 and a mean standard deviation of frequency > 0.08 kHz, as values with these parameters typically indicate false detections (Bliss 2020). Tags with these criteria were also examined individually using signal plots to identify false detections, which were manually removed from the dataset.

### 2.5 Length of Stay

We calculated the minimum length of stay (LOS) for individual birds (Anderson et al. 2019) that were detected at the two stopover sites during both migration periods. We refer to length of stay measurements as “minimums” because, for birds that were both tagged and detected within the same stopover site, it was not possible to determine the elapsed time between a bird's arrival at the site and the time it was tagged. For birds first detected at their capture location (hereafter: “locally tagged birds” [e.g., a bird that was both tagged and detected in Fraser River Estuary in the same migration season]), length of stay was calculated within each stopover site as the duration of time from tag deployment to the last detection. For birds tagged outside their detection location (hereafter: “non‐locally tagged birds” [e.g., a bird tagged in California but detected in Fraser River Estuary]), length of stay was calculated within each stopover site as the duration of time from first tag detection to last tag detection. Based on Western Sandpiper flight speeds of 1017 km per day (Iverson et al. 1996), non‐local birds with a length of stay < 1 h were classified as “flyovers” and removed from analysis, as this length of stay value indicates the bird likely transited the site without stopping.

### 2.6 Invertebrate Prey Availability

We collected sediment cores along 200–500 m transects to assess the availability of invertebrate prey for migrating shorebirds during both migration seasons in 2021. At the Fraser River Estuary, we collected sediment cores at 50, 200, 350, and 500 m distances from the high tide line along nine transects. At the Tofino mudflats, we collected sediment cores at 50, 200, and 350 m intervals from the high tide line along five transects during northward migration and four transects during southward migration. Beaches in Tofino were narrower and had distinct zones. Therefore, we collected samples at the wrack line (visible high tide line), intertidal zone, and swash zone (shoreline) along nine transects. We collected sediment cores during receding tides, although, due to differences in tide levels and habitat availability, we collected samples within 2 h of high tide at mudflat habitats and 2 h of low tide at the beaches (Tofino only).

Sediment cores were collected using a 5 × 10 cm corer (196.36 mL), sieved through a 0.5‐μm mesh, and invertebrates were counted and identified to the lowest possible order. Sediment samples from mudflat habitats were processed within 24 h, and samples from the beach habitat in Pacific Rim National Park near Tofino were processed in situ. A subset of invertebrates, as well as unidentified specimens, were preserved in ethanol for further classification.

### 2.7 Data Analyses

To compare length of stay between study sites, we used generalized linear models (GLM) for modeling northward and southward data. The length of stay data were positive‐only and positively skewed. Therefore, we used a gamma error distribution with a log‐link to fit the models (Kohlmann et al. 1999). We assessed multicollinearity of the predictor variables by inspecting the variance inflation factor score using the *vif* function in the package *car* (Fox and Weisberg 2019). We visually inspected the residuals of final models using the “resid” function in R to confirm homoscedasticity. RStudio (v. 2021.09.0) was used to perform all statistical analyses. An alpha level of 0.05 was used to denote statistical significance.

We performed a discriminant function analysis (DFA) to assign sex to individual Western Sandpipers using culmen and tarsus measurements after Stein et al. (2008). If tarsus data were missing to use in the DFA (*n* = 168), we used the field‐assigned sex based on bill length.

We compared morphological variation in Western Sandpipers between stopover site locations using linear regression models on morphological data. Wing (mm), tarsus (mm), culmen (mm), and mass (g) were response variables with stopover site location, sex, age, and migration direction as predictor variables. For wing measures, we noted directly the confound between site and measurement type (flattened vs. natural position). Sex was included as a predictor variable in all models as Western Sandpipers are sexually dimorphic with males being smaller than females (Stein et al. 2008) and may be under different selection pressures to reach breeding grounds. Age was included as a predictor variable to account for variability in size between adults and juveniles (O'Hara et al. 2006; Fernández and Lank 2007).

We modeled northward and southward LOS data separately, given juveniles are only present during the southward migration period. Therefore, we included age as a predictor variable in the southward model only. To test the effect of migration season on length of stay, we ran a separate model with only adult birds. The tagging location variable was not included in the southward migration model, as only one bird was detected during southward migration that was tagged outside its detection location. Since juvenile Western Sandpipers migrate southward approximately 1 month later than adults (Ydenberg et al. 2004; Hope et al. 2011), and timing of capture efforts differed between the two stopover sites, the “number of days since first tag” variable was calculated as the number of days since the first bird of each cohort was tagged (Linhart 2021) to control for capture date and seasonal variation in length of stay. For example, if August 1 was the first day a juvenile Western Sandpiper was tagged in Tofino during southward migration, August 1 began as day 1 for all subsequent southward juvenile Western Sandpipers tagged in Tofino of that year.

The full models for Western Sandpipers included individual minimum length of stay as the response variable and included mass, sex, tagging location (tagged within or outside detection site), detection location (Fraser River Estuary or Tofino), numbers of days since first tag, year as a factor, and an interaction between detection location and mass as the predictor variables. We did not consider a size‐adjusted measure of body weight given the wing length was not measured consistently between sites, and mass was scaled using the *scale* function in R to standardize the continuous variable. To test for differences in effects of mass between stopover sites, an interaction between mass and stopover site was included in all models. To account for differences between non‐locally tagged birds, the northward model included two‐way interactions between tagging location and mass and tagging location and days since first tag. To account for potential migration strategy differences between ages, the southward model included an interaction between age and days since first tag, and age and mass. Along with interactions listed above for each migration season, we modeled all possible combinations of significant predictor variables and compared Akaike's Information Criterion (AIC) values of each model to select the most parsimonious model. To explore significant interactions between variables, we used the emtrends() function from the *emmeans* package in R.

To assess invertebrate numbers between stopover sites and migration seasons, we used generalized linear models with a Poisson distribution (*glm* function in R). In these models we included the number of invertebrates per sample core (196.36 mL) as the response variable with stopover site and migration season as predictor variables.

## 3 Results

We tagged 395 Western Sandpipers from 2018 to 2021, including 179 birds at the Fraser River Estuary and 216 birds in Tofino. Ninety‐six percent (379/395) of the tagged Western Sandpipers were subsequently detected by receivers in their respective project arrays. An additional 107 birds were tagged in Mexico and California during northward migration, and 39 of these birds (36%) were subsequently detected in British Columbia. We removed birds with missing relevant attributes (i.e., mass, age, etc.) from models, which left a sample size of 385 Western Sandpipers (northward *n* = 149, southward *n* = 246) for analyses.

### 3.1 Length of Stay

**FIGURE 2:**
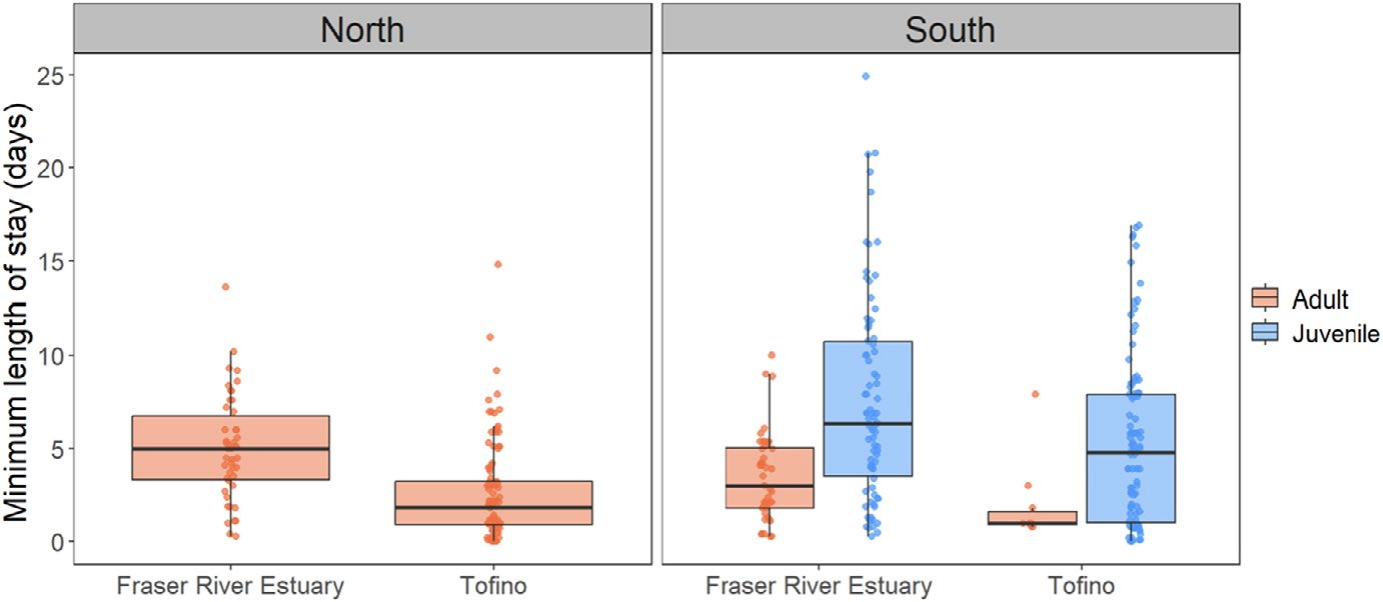
Minimum length of stay (LOS) of Western Sandpipers in the Fraser River Estuary and Tofino stopover sites during northward (Fraser River Estuary = 46, Tofino = 104) and southward (Fraser River Estuary adults = 44, Tofino adults = 10; Fraser River Estuary juveniles = 84, Tofino juveniles = 100) migration seasons from 2018 to 2021. LOS is calculated for each stopover site by subtracting the time of tag deployment from the time of last detection for birds that were both tagged and detected within the same stopover site, or by subtracting the time of first detection from the time of last detection for birds tagged outside the stopover site they were detected. The boxplot denotes the median and upper and lower quartiles of the data. The vertical lines extend to 1.5× the interquartile range.

**FIGURE 3:**
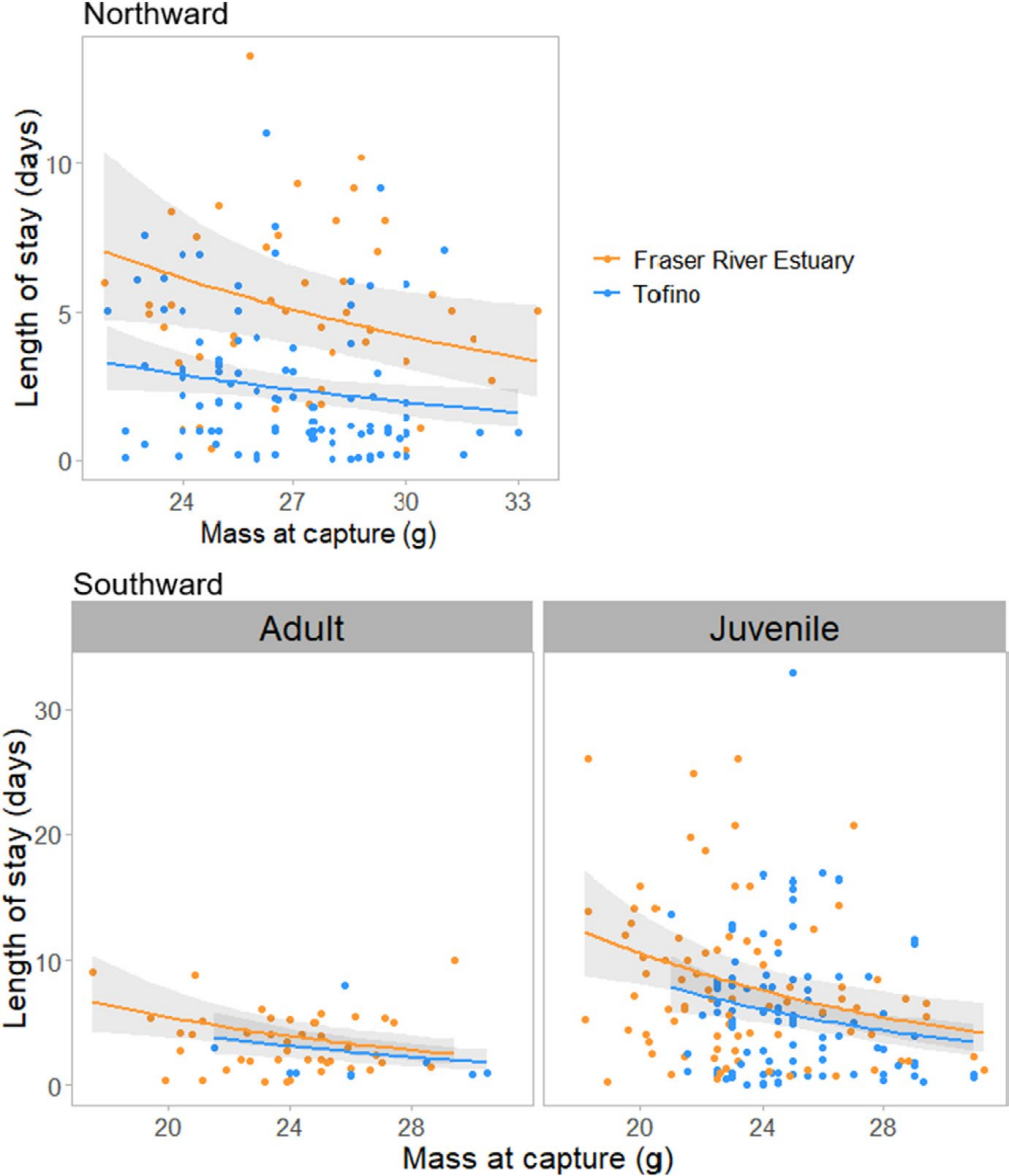
Relationship between mass and minimum length of stay in days of tagged Western Sandpipers by stopover site during northward migration (top), and by stopover site and age during southward migration (bottom). Data points are raw values, and fitted lines are predicted values with 95% confidence intervals.

As overall averages, the minimum length of stay (LOS) of tagged Western Sandpipers was 3.3 ± 0.2 days during northward migration and 6.1 ± 0.4 days during southward migration, and details varied among stopover sites, age classes, and migration periods (Table 2). The LOS varied significantly between stopover sites; juvenile and adult Western Sandpipers detected at the Tofino study site stayed for ~2.0 to 2.5 fewer days than Western Sandpipers at the Fraser River Estuary, respectively (Figure 2, Table 2). Length of stay was negatively correlated with body mass during both migration seasons, i.e., lighter birds stayed for longer (Figure 3, Table 3). During northward migration, non‐locally tagged Western Sandpipers had a significantly shorter LOS than birds that were captured and tagged within the study location (Table 3). There was a significant interaction between capture location and mass, where mass was negatively correlated with length of stay for locally tagged birds but not for non‐locally tagged birds. Males had significantly shorter stopovers than females during northward migration (Table 3). During northward migration, there was no significant effect of days since first tag, and no significant interactions between capture site and days since first tag or detection site and mass.

**TABLE 2:** Mean length of stay (LOS) in days of tagged Western Sandpipers by age and migration season in the Fraser River Estuary and Tofino migration stopover sites.

Migration direction	Age	Stopover site	Tagged (*n*)	Detected (*n*)	Minimum estimated LOS (Days ± SE)
Northward	Adult	Fraser River Estuary	45	46	5.0 ± 0.4
		Tofino	103	104	2.5 ± 0.3
Southward	Adult	Fraser River Estuary	47	44	4.4 ± 1.0
		Tofino	11	10	1.9 ± 0.7
Southward	Juvenile	Fraser River Estuary	84	84	7.9 ± 0.7
		Tofino	102	100	5.9 ± 0.6

*Note:* LOS is calculated for each stopover site by subtracting the time of tag deployment from the time of last detection for locally tagged birds, or by subtracting the time of first detection from the time of last detection for non‐locally tagged birds. Tagged (*n*) indicates the number of birds tagged locally at the site, and detected (*n*) indicates the total number of birds detected, including non‐locally tagged birds.

**TABLE 3:** Top Generalized Linear Model coefficients and results for minimum length of stay (LOS) of each model category (by migration season) selected using the lowest AIC values.

Model	df	Predictor variable	Estimate	Standard error	*t*	*p*
Northward	8	Capture site (Outside)	−1.35	0.28	−4.82	< 0.01
		Mass	−0.27	0.08	−3.40	< 0.01
		Sex (Male)	−0.41	0.16	−2.52	0.01
		Stopover site (Tofino)	−0.94	0.16	−5.80	< 0.01
		Capture site (Outside)*Mass	0.71	0.34	2.04	0.04
Southward	10	Age (Juvenile)	0.30	0.19	1.59	0.11
		Days since first tag	−0.06	0.22	−2.82	< 0.01
		Mass	−0.25	0.06	−4.03	< 0.01
		Stopover site (Tofino)	−0.62	0.17	−3.64	< 0.01
		Year (2019)	1.29	0.32	3.90	< 0.01
		Age (J) * Days since first tag	0.05	0.22	2.11	0.04
North + South	5	Migration direction (S)	−0.38	0.19	−2.00	0.048
		Mass	−0.21	0.08	−2.72	< 0.01
		Stopover site (Tofino)	−0.76	0.16	−4.80	< 0.01

*Note:* Minimum length of stay (LOS) in days is the dependent variable. Categorical predictor variables are capture site (captured within or outside detection site), days since first tag (capture day of year), stopover site (Tofino and Fraser River Estuary), sex, migration direction (Northward and Southward), and year; continuous predictors are mass (g; mass).

During southward migration, juveniles stayed longer than adults (Figure 2), and there was a significant interaction between days since first tag and age, where adults arriving earlier stayed longer (Figure 2, Tables 2 and 3). Western Sandpipers had a significantly longer stopover in 2019 during southward migration (Table 3). In the southward models, there were no interactions between detection site and mass, age class and mass, and there was no effect of sex. In the model combining both migration periods with only adult birds, adults stayed at the stopover sites significantly shorter during southward migration compared to northward migration (Figure 2, Tables 2 and 3).

### 3.2 Detections of Non‐Locally Tagged Birds

Thirty‐nine Western Sandpipers that were tagged at the southern non‐breeding sites of San Francisco Bay, California, and Sinaloa, Mexico, were detected by receivers in the project array in British Columbia. Two southern‐tagged birds were detected in Tofino during both northward and southward migration. Only 28% of these southern‐tagged birds were considered to have a stopover, where the remainder were deemed “flyovers” as their length of stay was < 1 h. Although most of the southern‐tagged birds were detected in Tofino during northward migration (tags detected in Tofino = 32, tags detected in the Fraser River Estuary = 7), these birds were also significantly less likely to stop in Tofino (19%), that is, more likely to overfly the site than the Fraser River Estuary, where the five detected birds were more likely to stop (71%) (*z* = −2.6, *p* < 0.01). Western Sandpipers tagged at southerly sites that stopped for > 1 h in Tofino stayed for 0.4 ± 0.2 days (*n* = 6), which was a shorter length of stay than birds that stopped in the Fraser River Estuary, 2.1 ± 0.7 days (*n* = 5). The northward length of stay of all southern‐tagged Western Sandpipers that were detected at our study sites, including birds classified as flyovers, was 0.08 ± 0.05 days in Tofino (*n* = 32) and 1.5 ± 0.6 days in the Fraser River Estuary (*n* = 7).

There were four instances (1.0% of tagged birds) of individual Western Sandpipers that were detected using both study stopover sites between and within migration seasons. In 2021, two birds tagged in the Fraser River Estuary during northward migration were subsequently detected in Tofino during the same migration period. During southward migration in 2021, two birds tagged in Tofino were subsequently detected in the Fraser River Estuary, one tagged during each migration season.

### 3.3 Morphology

The morphometrics of Western Sandpipers varied between the two stopover sites, migration seasons, and with sex. During southward migration, birds captured in Tofino were significantly heavier than birds captured in the Fraser River Estuary [FRE *n* = 127, Tofino *n* = 102; df (3225); *t* = 5.14, *p* < 0.01]. During northward migration, there were no significant differences in mass between sites [FRE *n* = 39, Tofino *n* = 102; df (2138); *t* = −0.8, *p* = 0.4]. Males were also significantly lighter than females during both migration periods [Northward: female *n* = 61, male *n* = 80; df (2138); *t* = −4.96; *p* < 0.01; Southward: female *n* = 104, male *n* = 125; df (3, 225); *t* = −7.04; *p* < 0.01] (Table 4). Males are known to be smaller than females (Stein et al. 2008).

**TABLE 4:** Average length of culmen, tarsus, wing length, and mass of Western Sandpipers by stopover site, sex, and migration direction.

Feature	Sex	Northward migration				Southward migration							
		Adults				Adults				Juveniles			
		Fraser River Estuary		Tofino		Fraser River Estuary		Tofino		Fraser River Estuary		Tofino	
		*n*	Length ± SD (mm)	*n*	Length ± SD (mm)	*n*	Length ± SD (mm)	*n*	Length ± SD (mm)	*n*	Length ± SD (mm)	*n*	Length ± SD (mm)
Culmen	Female	15	26.9 ± 0.76	46	26.8 ± 1.05	19	26.9 ± 1.05	4	26.5 ± 0.74	40	26.4 ± 0.69	30	26.8 ± 0.93
	Male	24	22.6 ± 0.88	56	22.6 ± 1.12	25	22.8 ± 1.01	4	22.0 ± 0.48	44	22.6 ± 1.21	43	22.4 ± 1.20
Tarsus	Female	7	26.4 ± 0.56	13	23.9 ± 0.88	19	26.3 ± 1.05	1	24.4	39	25.5 ± 1.42	9	23.8 ± 0.55
	Male	12	23.8 ± 1.67	25	22.4 ± 0.87	24	24.5 ± 1.26	1	23.5	44	23.9 ± 1.31	24	22.4 ± 1.04
Wing	Female	15	103 ± 2.26	45	99.6 ± 1.96	19	100 ± 3.81	4	96.6 ± 5.50	40	101 ± 1.70	36	100. ±2.88
	Male	21	98.5 ± 2.18	54	95.7 ± 2.17	25	97.7 ± 1.99	4	95.8 ± 1.71	43	98.3 ± 2.29	46	96.6 ± 2.42
Mass	Female	15	29.1 ± 2.22	46	27.7 ± 2.42	19	25.5 ± 1.84	5	27 ± 2.49	40	24.4 ± 2.94	40	26.0 ± 2.40
	Male	24	25.8 ± 2.11	56	26.2 ± 2.31	24	22.5 ± 2.27	4	24.4 ± 2.14	44	22.3 ± 2.72	53	24.1 ± 1.62

*Note:* Average mass (g) of Western Sandpipers by stopover site, age, and migration direction. Measurements were acquired at capture from 2018 to 2021. Wing measurements were recorded as “wing chord” in Tofino and “wing chord” and “flattened wing” in the Fraser River Estuary.

**FIGURE 4:**
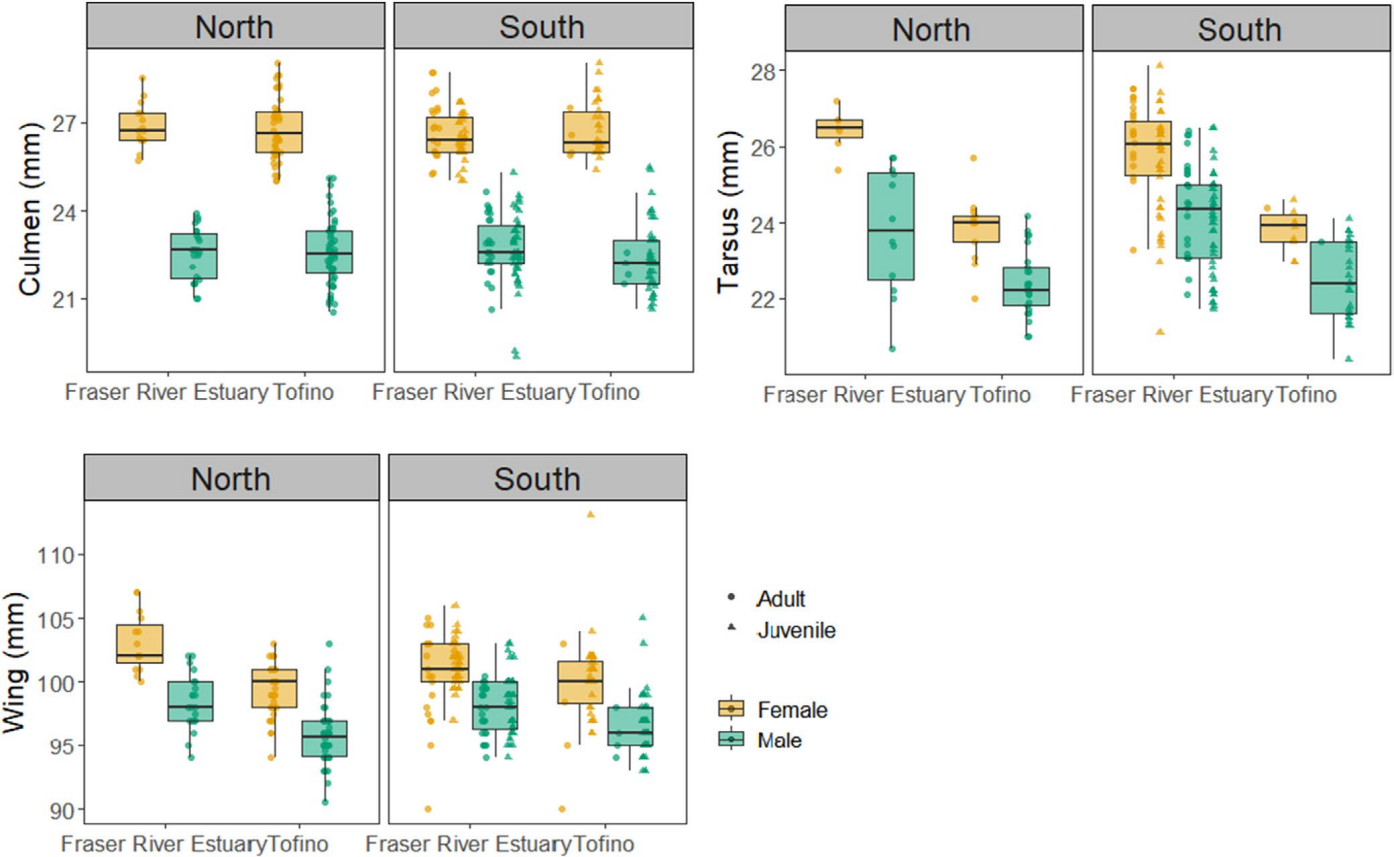
Culmen (mm), tarsus (mm), and wing (mm) of tagged Western Sandpipers by migration direction, age, and sex in the Fraser River Estuary and Tofino stopover sites. Wing measurements were recorded as “natural wing chord” in Tofino and “natural wing chord” or “wing flattened” in the Fraser River Estuary. Boxplots represent raw data and morphometric variables, and tarsus and wing measurements had significant differences between stopover sites. The boxplot denotes the median and upper and lower quartiles of the data. The vertical lines extend to 1.5× the interquartile range.

Western Sandpipers that were captured in Tofino also had significantly shorter tarsi (mm) [df (5, 215); Location (Tofino): *t* = −8.39; *p* < 0.01; Sex (Male): *t* = −10.08, *p* < 0.01; Age (Juvenile): *t* = −3.21, *p* < 0.01], as well as wing lengths (mm) [df (6, 362); Location (Tofino): *t* = −7.98, *p* < 0.01; Sex (Male): *t* = −13.35, *p* < 0.01; Age (Juvenile): *t* = 3.50, *p* < 0.01] than birds captured in the Fraser River Estuary (Figure 4). We note the differences in how wing measures were taken would account for differences in wing length between the two stopover sites. There was no significant difference in culmen lengths between the two stopover sites [df (6, 360); Location (Tofino): *t* = −0.54, *p* = 0.59; Sex (Male): *t* = −37.47, *p* < 0.01]. During southward migration, tarsi length was significantly longer than during northward migration (*t* = 2.14, *p* = 0.03), and wing length was significantly shorter (*t* = −2.88, *p* < 0.01) (Table 4).

### 3.4 Invertebrate Prey Abundance

We collected and processed a total of 216 sediment cores in the Fraser River Estuary and 222 sediment cores in Tofino. Overall invertebrate densities (number per 196.36 mL sample core) were nearly identical between the two sites (mean invertebrates per 196.36 mL; Tofino = 12.34 ± 1.2, Fraser River Estuary = 12.29 ± 0.9). However, seasonal differences did occur, and Tofino had a significantly higher density of invertebrates than the Fraser River Estuary during northward migration (FRE = 7.1 ± 0.6, Tofino 9.9 ± 1.3 mean invertebrates per 196.36 mL; *z* = 7.08, *p* < 0.01), and significantly lower density during southward migration (FRE = 17.5 ± 1.6, Tofino 15.1 ± 2.0 mean invertebrates per 196.36 mL; *z* = −4.3, *p* < 0.01). Migration season had an effect on invertebrate numbers, where there was a significantly overall higher invertebrate density during southward migration than northward migration at both the Fraser River Estuary (*z* = −21.02, *p* < 0.01) and Tofino (*z* = −10.09, *p* < 0.01) (Table 5).

**TABLE 5:** Percent composition and total number of invertebrates (*N*) from sediment core samples collected in the Fraser River Estuary and Tofino stopover sites during northward and southward migration in 2021.

Phylum	Class	Order	Northward				Southward			
			Fraser River Estuary		Tofino		Fraser River Estuary		Tofino	
			%	*N*	%	*N*	%	*N*	%	*N*
Annelida	Clitellata	Oligochaeta	0.4	3	5.4	62	0.1	1	0	0
	Polychaeta	Spp.	50.4	387	34.1	395	42.2	796	33.8	535
	Unknown	Spp.	17.2	132	2.8	32	16.8	318	2.1	33
Arthropoda	Copepoda	Spp.	0.3	2	0	0	0	0	0.1	1
	Insecta	Diptera	0	0	0.8	9	0.4	7	0.3	4
	Malacostraca	Amphipoda	6.9	53	41.9	485	32.1	606	48.3	764
		Cumacea	11.7	90	0	0	1.3	25	0	0
		Euphausiacea	0	0	0	0	0	0	0.9	14
		Isopoda	0	0	0.6	7	0	0	11.9	188
		Tanaidacea	1.3	10	10.3	119	0.5	9	0.2	3
Molluska	Bivalvia	Spp.	11.1	85	2.9	34	5.2	99	2.4	38
	Gastropoda	Spp.	0	0	0	0	0.6	12	0	0
Unknown	Unknown	Unknown	0.8	6	1.2	14	0.8	15	0.2	3
Total number of sediment cores sampled			108		117		108		105	
Average number of invertebrates per sample core			7.1 ± 0.6		9.9 ± 1.3		17.5 ± 1.6		15.1 ± 2.0	

*Note:* The same habitats in each stopover site were sampled during both migration periods.

## 4 Discussion

Stopover ecology of Western Sandpipers along the Pacific Americas Flyway has been well studied, particularly during the northward migration season (Iverson et al. 1996; Warnock and Bishop 1998; Williams et al. 2007; Hope et al. 2018). Fewer studies exist to track and compare strategies of Western Sandpipers during both northward and southward migrations or assess how migration strategies may differ (Duijns et al. 2019; Hope et al. 2011). We show that the length of stay of Western Sandpipers varied between stopover locations, migration seasons, and with age. Adults had longer stopovers during northward migration compared to southward migration, supporting the idea that adult Western Sandpipers are more time constrained during northward migration relative to the southward migration (Nilsson, Bäckman, and Alerstam 2014). Additionally, Western Sandpipers had shorter stopovers in Tofino compared to the Fraser River Estuary during both migration periods; juveniles had longer stopovers compared to adults, and LOS depended on mass at capture, indicating a condition‐dependent stopover decision.

### 4.1 Differences Between Stopover Sites

Western Sandpipers in both age groups and migration periods had a significantly shorter LOS at Tofino than at the Fraser River Estuary. Frequent, shorter stopovers are associated with a slower and safer migration strategy (Hope et al. 2011). Western Sandpipers are hypothesized to decrease their length of stay with increased predator presence or higher predation risk (Ydenberg et al. 2004; Pomeroy 2006). At low tide, beaches in Tofino are approximately 200 m wide, and the furthest distance to cover at the Tofino Mudflats is approximately 300 m. In the Fraser River Estuary, the mudflats are more expansive where the lowest tide mark can be over 5 km from shore (Government of British Columbia, GeoBC Branch 2020). Thus, the potentially greater predation danger at the Tofino site due to narrower site widths may result in the shorter length of stay at this site. However, sandpipers using stopover sites with greater predation danger also have lighter mass (Ydenberg et al. 2004), which allows for greater escape maneuverability (Burns and Ydenberg 2002). This finding does not align with our results during southward migration, where Western Sandpipers from Tofino were heavier than those at the Fraser River Estuary. We need independent measures of predation risk (e.g., counts of predators) to further assess the link between LOS and site characteristics.

The difference in LOS during southward migration between our study sites may result from differences in the mass of the birds at capture. In our study, adult and juvenile Western Sandpipers in the Fraser River Estuary were significantly lighter than birds in Tofino during southward migration, and during both migration periods, as mass increased, LOS decreased. These results support findings from previous studies that indicate sandpipers with lower mass, body condition, and/or smaller fuel reserves stay at sites longer than heavier birds (Skagen and Knopf 1994; Duijns et al. 2019; Herbert, Mizrahi, and Taylor 2022). However, this result contrasts with other sandpiper studies that found no relationship between body condition and length of stay (Iverson et al. 1996; Warnock and Bishop 1998; Linhart et al. 2023) and does not necessarily explain the longer length of stay at Fraser River Estuary during northward migration when there was no significant difference in mass of birds captured between sites.

The estimated minimum length of stay of Western Sandpipers in the Fraser River Estuary, derived from automated telemetry in this study (5.0 days northward and 4.5 days southward), is longer than mean estimates from previous studies conducted in the 1980s and 1990s from 1 to 3.6 days for northward and 3 days southward (Butler, Kaiser, and Smith 1987; Iverson et al. 1996; Warnock and Bishop 1998). These previous values are closer to the LOS of non‐locally tagged birds in our study, and therefore the longer average LOS in our study may have resulted from trends in behavioral changes over time or may be the result of a capture/handling effect. On average, locally tagged birds at the Fraser River Estuary and at Tofino stayed longer than the non‐locally tagged birds tagged south of BC (Fraser River: 5.0 vs. 2.1 days; Tofino: 2.6 vs. 0.4 days). Previous shorebird studies with Western Sandpiper and Dunlin have shown handling effects (Warnock and Bishop 1998; Warnock, Takekawa, and Bishop 2004). In contrast, Butler, Shepherd, and Lemon (2002) compared the LOS of Western Sandpipers at the Fraser River Estuary during northward migration between birds tagged locally with 0.8 g transmitters (*n* = 11) with birds similarly tagged in California (*n* = 4), and they found that average LOS was 2.8 days for both groups, albeit with small sample sizes. Skagen and Knopf (1994) found little effect of handling time on LOS of Semipalmated Sandpipers *Calidris pusilla* (a species like Western Sandpiper) fitted with 0.75 g transmitters (*n* = 35) during northward migration in Kansas, USA. This ambiguity about potential effects of handling birds means that we cannot infer about changes in LOS over time when comparing with previous studies and that more comprehensive analyses are needed to evaluate changes in LOS over time that account for both capture effects and changes in technology (manual vs. automated tracking).

### 4.2 Stopover Site Quality

Variation in LOS and mass of Western Sandpipers between the Fraser River Estuary and Tofino suggests a difference in site quality between the two stopover sites. Overall, shorebirds have longer lengths of stay at high‐quality stopover sites with a high abundance of prey (Warnock, Takekawa, and Bishop 2004; Herbert, Mizrahi, and Taylor 2022). The compositions of habitats at our two study sites offer variable prey selection for Western Sandpipers, and Tofino had a significantly higher density of invertebrates during northward migration compared to the Fraser River Estuary and lower densities during southward migration. During southward migration, birds that stopped at the Fraser River Estuary were lighter and had a longer LOS than birds in Tofino. While this difference might suggest that the higher density of invertebrates at the Fraser River Estuary during southward migration could drive the longer length of stay, LOS was consistently longer at the Fraser River Estuary compared to Tofino during both migration periods, even with prey density varying between migrations at each site. Therefore, invertebrate numbers alone are unlikely to explain differences in LOS between sites.

Intertidal biofilm is an important component of the Western Sandpiper diet (Elner et al. 2005; Kuwae et al. 2008; Jardine et al. 2015; Hobson et al. 2022). Studies showed that the characteristics of the quiescent, estuarine mudflat habitats that make up the Fraser River Estuary are ideal conditions for intertidal biofilm (de Jorge and van Beusekom 1995; Kuwae et al. 2008), compared to less favorable sandy substrates that comprise much of the Tofino study sites. Lipid content in intertidal biofilm is associated with high use of mudflats by migrating shorebirds (Drever et al. 2024). Preliminary results suggest that the fatty acid content of surface sediment (a marker of site quality) on the Tofino mudflats is lower than that of Roberts Bank in the Fraser River Estuary (Drever et al. 2023, unpublished data). Given the longer length of stay and lighter mass (southward) of Western Sandpipers in the Fraser River Estuary, birds who stop at this site may be arriving after a long flight to target the essential fatty acids of both biofilm and invertebrate prey, thus staying longer to maximize fueling rates and gain higher fat stores (Hope et al. 2011; Schnurr et al. 2020). Conversely, southward Western Sandpipers who stop in Tofino may be arriving after a shorter flight from a nearby stopover site, and thus do not require high fueling rates, and are able to continue onwards with short flights with sufficient fat stores from the availability of amphipods and large polychaetes (e.g., *Mucronata* spp.) found at this site.

### 4.3 Migration Routes and Strategies

Few individual birds in our study were detected in both the Fraser River Estuary and Tofino stopover sites, whether within or between migration seasons, suggesting that individuals tend to stop at either site but not both. Western Sandpipers that migrated northward from California and Mexico were more likely to be detected in Tofino compared to the Fraser River Estuary. Most of these southern‐tagged birds flew by Tofino, and if they stopped, did so for brief periods. Although fewer southern‐tagged Western Sandpipers were detected in the Fraser River Estuary, a greater proportion of birds that were detected at this location stopped for a longer duration, suggesting they were actively refueling. Migrating along the outer west coast of Vancouver Island may offer a more direct route for Western Sandpipers flying between their west coast wintering (non‐breeding) grounds and Arctic breeding grounds in Alaska (Figure 1). Since birds captured at the Tofino stopover had a higher mass during southward migration, it is possible that birds in better condition selected the more direct migration route along the outer coast. As the Fraser River Estuary represents a slight eastbound detour for Western Sandpipers (Figure 1), birds that select this stopover may trade off the increased distance to target the site for specific attributes of intertidal biofilm (Schnurr et al. 2019, 2020).

The difference in stopover length between sites could be a result of differing populations and migration strategies. Shorter stopovers are associated with a slower migration strategy, both characteristics of “hop” migrants who fly short‐to intermediate distances between stopover sites. The estimated peak passage of Western Sandpipers in Tofino (May 3) is approximately 4 days later than those birds stopping at the Fraser River Estuary (April 29) (Hope et al. 2018). Western Sandpipers that stop in Tofino may be using more of a “hop” migration strategy than birds who stop at the Fraser River Estuary, and as suggested by their later peak (northward) arrival time and shorter stopover duration, may travel shorter migration distances than birds who stop at the Fraser River Estuary. It is possible that birds who stop in Tofino arrive from closer non‐breeding sites and/or fly to closer breeding sites. However, further studies assessing Western Sandpiper lengths of stay and morphology among multiple sites along the Pacific Americas Flyway would be required for inferences regarding population partitioning between sites on the outer Vancouver Island coast and the Strait of Georgia.

**FIGURE 5:**
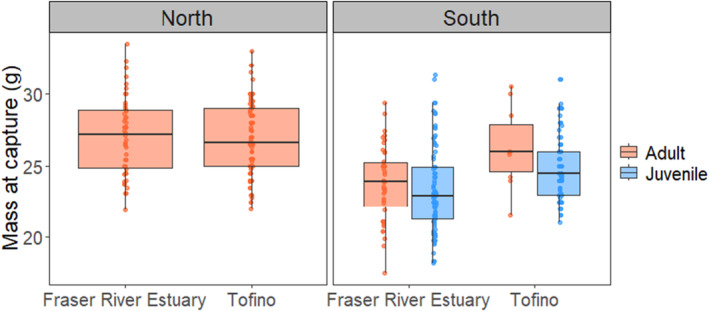
Mass (g) by migration direction and age of tagged Western Sandpipers at the Fraser River Estuary and Tofino stopover sites. Boxplots represent raw data, and variables had significant statistical differences between stopover sites. The boxplot denotes the median and upper and lower quartiles of the data. The vertical lines extend to 1.5× the interquartile range.

Western Sandpipers stopping in Tofino were smaller than Western Sandpipers that stop in the Fraser River Estuary (Figure 5). Genetic differences among Western Sandpiper populations are unclear (Haig et al. 1997; Alvarez Sánchez 2011), and there is evidence of low levels of population structure (Schwarz 2016), including morphological and sexual segregation on their wintering grounds (Nebel et al. 2002; O'Hara et al. 2006). O'Hara et al. (2006) found that, within sexes, Western Sandpipers that spend the non‐breeding period in southern latitudes are larger in wing chord and culmen than Western Sandpipers that spend this season at more northerly latitudes. Isotopic evidence suggests that Western Sandpipers at the Fraser River Estuary come from a wide variety of non‐breeding areas (Franks et al. 2012). We found Western Sandpipers at Tofino had significantly shorter wing lengths than at the Fraser River Estuary, although we cannot make many inferences based on these differences, given the inconsistencies in wing measurement methods at the Fraser River Estuary. With the caveat that allometric scaling may also explain these results, we note that differences in wing lengths were matched by differences in tarsi, where birds at Tofino had smaller tarsi, indicating birds at Tofino may represent a smaller subset of the population coming from relatively more northerly non‐breeding areas. This interpretation is also consistent with the pattern of shorter stopovers at the Tofino study area.

### 4.4 Southward Migration: Age and Predation Danger

Western Sandpiper adults and juveniles have similar migration patterns in both stopover sites during southward migration—adult Western Sandpipers have shorter LOS compared to northward migration, and juveniles have a longer LOS compared to adults during southward migration. The longer stopover of juveniles compared to adults supports findings from previous studies on Semipalmated Sandpipers (Roques et al. 2021; Linhart et al. 2023). Previous studies assessing Western Sandpiper length of stay in both age groups during southward migration in the Fraser River Estuary or nearby sites found that adults stayed for around 3 days (Butler, Kaiser, and Smith 1987), whereas juveniles stayed from 3.8 to 12 days (Hope et al. 2011, 2021; Drever and Hrachowitz 2017), a difference that was similar to our study. During southward migration, adult Western Sandpipers leave the breeding grounds early such that their migration precedes the Peregrine Falcon migration that occurs in late July (Ydenberg et al. 2004; Hope et al. 2011). Juveniles remain in their breeding grounds longer than adults to continue to feed (Franks, Lank, and Wilson Jr. 2020), putting them at increased predation risk during their southward migration that occurs concurrently with Peregrine Falcon migration (Lank et al. 2003). Since juveniles experience a riskier migration than adults, a cautious migration strategy with more frequent, shorter stopovers would be expected (Hope et al. 2011), which was not seen in our study. However, juveniles may increase vigilance while foraging, which can lead to reduced fuel loading rates, and, in turn, can decrease wing loading and improve escape performance from predators (Burns and Ydenberg 2002; Ydenberg et al. 2004; Hope et al. 2011; Hope, Lank, and Ydenberg 2014). As juveniles may be using more cautious behaviors during stopover than adults, along with their inexperience in migration, it is possible their behaviors lengthen stopover duration as they require more time to gain fat stores (Duijns et al. 2019).

Adult Western Sandpipers that arrived earlier at their stopover sites stayed longer in our study. As longer stopovers are associated with faster overall migrations (Piersma 1987; Warnock 2010), it would be expected that stopover length would increase with later arrival times to stay ahead of the arriving falcon front (Hope et al. 2011; Anderson et al. 2019). Studies on migrating Western Sandpipers at the Fraser River Estuary have shown seasonal speed adjustments during southward migration, using a “caution–speed–caution” strategy. Sandpipers arriving well ahead of the falcon migration can afford a slower and more cautious migration strategy, and as the “falcon front” approaches, migration speed increases with longer lengths of stay. The latest arriving sandpipers that are at greatest risk of predation once again slow their speed of migration, where stopover time is reduced (Hope, Lank, and Ydenberg 2014; Ydenberg et al. 2004). Adult Western Sandpipers captured earlier in our study may have coincided with the “speed” strategy, and later arriving adults who were at greatest risk of predation used a more cautious strategy. As lighter birds have improved escape performance from predators, later arriving adults at greater risk of encountering falcons may have reduced their stopover time to decrease fuel loads and maintain lower wing loading. Since we did not record predator presence in our study, it is unknown whether later arriving adult Western Sandpipers coincided with the arrival of falcons. However, the differences in stopover length between juveniles and adults, as well as the interaction between arrival time and stopover length of adults, suggest that Western Sandpipers may also be using a mortality‐minimizing strategy during southward migration.

## 5 Conclusions

The minimum length of stay of migrating Western Sandpipers at two stopover sites in the Pacific Americas Flyway varied depending on migration strategy, season, individual biology, site characteristics, and location. Variation in morphology between sites suggests individuals from different overwintering populations may use different routes along the west coast of North America and use stopover sites based on predation risk, food availability, and their own condition. Different stopover sites offer a unique set of characteristics used by birds exhibiting varying migration strategies. The differences in LOS between adults and juveniles mean that surveys may need to vary in frequency between July and August to avoid double‐counting birds (Crewe, Lepage, and Taylor 2016). In addition, sandpipers in our study stopped at either the Fraser River Estuary or Tofino and did not fly between both, which highlights the importance of conserving both larger stopover sites like the Fraser River Estuary as well as smaller sites like those around Tofino (Linhart et al. 2023). Our results also suggest that a more detailed understanding of population structure and connectivity within the Pacific Americas Flyway would likely support a more accurate assessment of population trends for this species.

## Author Contributions

**Anne L. Blondin:** data curation (equal), formal analysis (equal), software (equal), visualization (equal), writing – original draft (equal). **Mark C. Drever:** conceptualization (equal), funding acquisition (equal), investigation (equal), project administration (equal), supervision (equal), writing – review and editing (equal). **Scott A. Flemming:** funding acquisition (equal), investigation (equal), methodology (equal), resources (equal), supervision (equal), writing – review and editing (equal). **Wendy E. Easton:** conceptualization (equal), funding acquisition (equal), investigation (equal), project administration (equal), writing – review and editing (equal). **Mark Maftei:** conceptualization (equal), funding acquisition (equal), investigation (equal), writing – review and editing (equal). **Yuri Zharikov:** conceptualization (equal), funding acquisition (equal), investigation (equal), writing – review and editing (equal). **Nils Warnock:** investigation (equal), methodology (equal), resources (equal), writing – review and editing (equal). **Erica Nol:** project administration (equal), supervision (equal), writing – review and editing (equal).

## Conflicts of Interest

The authors declare no conflicts of interest.

## Data Availability

Shorebird detection data is available through the data portal at https://motus.org/. Processed detection data will be made available through Dryad.
